# Two new *Neuratelia* Rondani (Diptera, Mycetophilidae) species from Western Palaearctic: a case of limited congruence between morphology and DNA sequence data

**DOI:** 10.3897/zookeys.496.9315

**Published:** 2015-04-16

**Authors:** Olavi Kurina, Erki Õunap, Kadri Põldmaa

**Affiliations:** 1 Institute of Agricultural and Environmental Sciences, Estonian University of Life Sciences, Kreutzwaldi st 5D, 51014 Tartu, ESTONIA; 2 Department of Zoology, Institute of Ecology and Earth Sciences, University of Tartu, Vanemuise 46, 51014 Tartu, ESTONIA; 3 Department of Botany, Institute of Ecology and Earth Sciences, University of Tartu, Ravila 14a, 50411 Tartu, ESTONIA

**Keywords:** Mycetophilidae, *Neuratelia*, new species, Western Palaearctic, systematics, molecular analysis, COI, ITS2, 28S

## Abstract

Two new Mycetophilidae species, *Neuratelia jabalmoussae* **sp. n.** and *Neuratelia salmelai* **sp. n.** are described on the basis of material collected from Lebanon, Estonia and Finland. Detailed figures of male terminalia and photographs of general facies are provided along with discussions of their morphological distinction from sibling species. For the first time molecular characters are used to distinguish new fungus gnat species. Molecular analysis relies on cytochrome oxidase subunit one (COI) but has additionally been corroborated by information from the 28S and ITS2 regions of nuclear ribosomal DNA. Situations where morphological and molecular data provide conflicting evidence for species delimitation are discussed. A new country record from Georgia is provided for *Neuratelia caucasica*.

## Introduction

The genus *Neuratelia* Rondani, 1856 forms a well delimited clade in the subfamily Sciophilinae (Mycetophilidae), as sister group to the remaining Sciophilinae (Borkent & Wheeler, 2013). According to Søli et al. (2000) it is characterised by the following combination of characters: laterotergite setose, M and CuA clearly branched but base of M_1_ obsolete, R_5_ strongly sinuate, C produced about one fifth of the distance between apex of R_5_ and apex of M_1_, and tibia with distinct setae. Very little is known about their biology; according to Laffoon (1965) the larvae of one species were found in moss. This is, however, challenged by Hutson et al. (1980). Altogether 31 extant species are known from across the world including 16 species from the Palaearctic region (Matile 1974, Zaitzev 1994, Sasakawa 2004), 13 species from the Nearctic region (Borkent and Wheeler 2013) and one from both the Neotropical and Oriental regions (Bechev 2000). Additionally, three species have been described from fossils (Evenhuis 2014). Among the Palaearctic species seven are so far known to occur in the Western Palaearctic. There are no keys to cover all described species of the world, of only the Palaearctic region or even just in Europe. For Western Palaearctic species, the most exhaustive one is the key by Zaitzev (1994) that excludes, however, several European species.

So far, alpha-taxonomy of fungus gnats has been carried out using traditional taxonomic methods, primarily morphological examination. Though in recent years nucleotide data have been implemented to address the phylogeny of this group (e.g. Rindal et al. 2009a, 2009b, Ševčík et al. 2013, 2014), to associate sexes of one species (Kurina et al. 2011) and in population genetic studies (Dörge et al. 2014). Hippa and Ševčík (2014) provided mitochondrial 12S and 16S sequences in the description of *Nepaletricha sigma*. Despite that, no molecular information has so far been utilised for delimitation of a new fungus gnat species. This is surprising, as using a 658-bp fragment from the 5’ end of the mitochondrial cytochrome oxidase gene subunit 1 (COI) – the so-called ‘DNA barcode’ (see Hebert et al. 2003) – has become an increasingly common practice in discriminating insect species during recent years (e. g. Yassin 2008, Huemer and Hebert 2011, Riedel et al. 2013). Sometimes, acquiring additional genetic data from other loci has also been used to corroborate findings discovered by studying DNA barcodes (e.g. Õunap and Viidalepp 2009, Raupach et al. 2010, van Nieukerken et al. 2012).

The aim of this article is to publish taxonomic and faunistic information about Western Palaearctic *Neuratelia* specimens that the senior author has accumulated over recent years. Both morphological and molecular data were used for species delimitation. This resulted in describing two new species – one from Estonia and Finland and another from Lebanon.

## Material and methods

### Collection, preparation, illustration and morphological study

**Figures 1–3.**
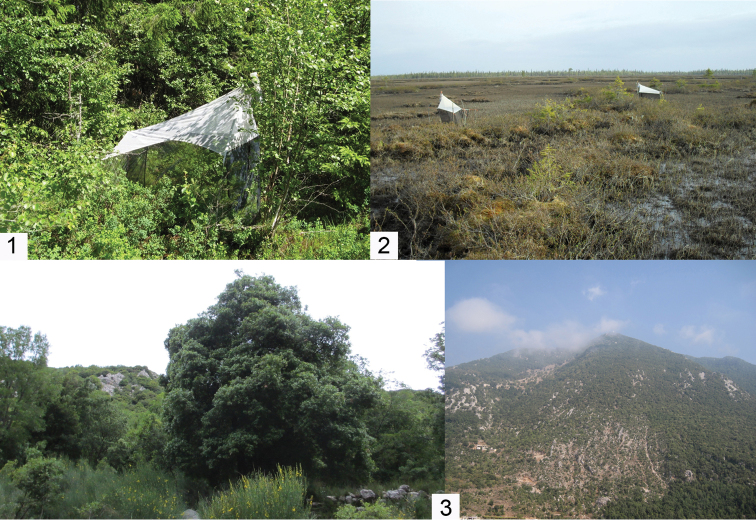
Collecting localities of *Neuratelia salmelai* sp. n. (**1, 2**) and *Neuratelia jabalmoussae* sp. n. (**3**). **1** Palupõhja in Estonia (holotype) **2** Kaita-aapa (Sodankylä) in Finland (a paratype) **3** Mar Elias in Jabal Moussa Biosphere Reserve, Lebanon (holotype).

The examined material of two new species was collected from Estonia and Finland using Malaise traps, and from Lebanon by light trapping, respectively. The Estonian locality lies at the herb rich edge of a mixed forest (Fig. 1) while the Finnish localities are predominantly wet fen habitats (Fig. 2) with variable vegetation irrigated by occasional springs. All Finnish localities are from the northern part of the country. In Lebanon, the material was collected from Jabal Moussa Biosphere Reserve, north-east of Beirut, characterised by karstic mountains with evergreen sclerophyllous vegetation (Fig. 3). The additional studied *Neuratelia* material was collected from Georgia, Greece, Slovakia, Finland and Estonia by sweep netting and Malaise trapping.

All specimens were stored initially in ethyl alcohol within which parts of them – after studying under a stereomicroscope Leica S8APO – are still preserved. For more detailed study of male terminalia, they were detached and macerated in a 10% solution of KOH, followed by neutralization and washing in distilled water. The remaining chitinous parts were thereafter inserted into glycerine for study, including black and white illustrations, and preserved as glycerine preparations in polyethylene microvials (see also Kurina 2003). A few specimens including their terminalia were slide mounted in Euparal following the method described by Hippa and Kurina (2012). The current preservation method of each specimen is indicated in the material section. The measurements are given as the range of measured specimens followed by the mean value, while measurements from the holotypes are given in square brackets. The ratios of the three apical palpal segments are given as 3^rd^:4^th^:5^th^. All measurements are taken from specimens in alcohol. Morphological terminology follows Søli et al. (2000).

The habitus photos have been made in an alcohol medium using a Canon 7D camera with a Canon MP-E65 (F2.8 1–5×) lens (see Kurina et al. 2011). The photos of thorax and terminalia were combined using the software LAS V.4.1.0. from multiple gradually focused images taken by a Leica DFC 450 camera attached to a Leica 205C stereomicroscope or Leica DM 6000 B compound microscope, respectively. Adobe Photoshop CS5 was used for editing the figures and compiling the plates. Black and white illustrations of the terminalia were prepared using a U-DA drawing tube attached to an Olympus CX31 compound microscope.

The material is deposited in the Institute of Agricultural and Environmental Sciences, Estonian University of Life Sciences [former Institute of Zoology and Botany], Tartu, Estonia (IZBE), in the Zoological Museum, University of Turku, Finland (ZMUT) and in the personal collection of J. Salmela, Rovaniemi, Finland (JSPC).

### Molecular techniques

The genomic DNA was extracted using High Pure PCR Template Preparation Kit (Roche Diagnostics GmbH, Mannheim, Germany). Anterior segments of the abdomen that had been stored after dissection of genitalia were crushed and used for the extraction. This process was carried out following the manufacturer’s instructions for extraction of genetic material from mammalian tissue.

In total, one mitochondrial and two nuclear markers were sequenced. A 658-bp ’barcoding’ fragment from close to the 5’ terminus of the mitochondrial gene cytochrome C oxidase subunit 1 (*COI*), was amplified and sequenced using primers LCO1490 (5’-GGT CAA CAA ATC ATA AAG ATA TTG G-3’) and HCO2198 (5’-TAA ACT TCA GGG TGA CCA AAA AAT CA-3’) (Folmer et al. 1994). A 695-701-bp fragment covering expansion segments D1 and D2 of the nuclear 28S rRNA gene was sequenced using primers D1F (5’-GGG GAG GAA AAG AAA CTA AC-3’) (Abraham et al. 2001) and D2R (5’-TTG GTC CGT GTT TCA AGA CGG G-3’) (Belshaw and Quicke 1997). In the case that this preferable treatment was not successful, the desired part of the 28S was sequenced in two fragments, combining D1F with D1R (5’-CAA CTT TCC CTT ACG GTA CT-3’) (Abraham et al. 2001) and D2R with D2F (5’-AGA GAG AGT TCA AGA GTA CGT G-3’) (Belshaw and Quicke 1997). In addition, a fragment of the internal transcribed spacer 2 region (ITS) located between the 5.8S rRNA and 28S rRNA genes was sequenced using primers ITS2A (5’-TGT GAA CTG CAG GAC ACA T-3’) and ITS2B (5’-TAT GCT TAA ATT CAG GGG GT-3’) (Beebe and Saul 1995). PCR was performed in a total volume of 25 µl, with the reaction mixture containing 1X HOT FIREPol® Blend Master Mix Ready to Load (Solis BioDyne, Tartu, Estonia), 10 pmol of primers and 20-80 ng of purified genomic DNA. PCR was carried out in an Eppendorf Mastercycler epigradient thermocycler (Eppendorf AG, Hamburg, Germany). Its conditions involved an initial denaturation at 95 °C for 15 min, 35 cycles of 30 s at 95 °C, 30 s at 45–60 °C (depending on primers) and 1 min at 72 °C, followed by a final extension at 72°C for 10 min. PCR products were visualised on a 1.2% agarose gel, and 20 μl of the PCR solution was treated with fast alkaline phosphatase and exonuclease I (Thermo Scientific, Pittsburgh, USA). In some cases, direct sequencing from PCR solution was not possible due to multiple products. To sequence these samples, desired products were cut from agarose gel and extracted using a High Pure PCR Product Purification Kit (Roche). DNA cycle sequencing was performed either by Macrogen Europe (Amsterdam, Netherlands) or by the Estonian Biocentre (Tartu, Estonia). Both DNA strands were sequenced for all studied markers.

### Phylogenetic analysis

Consensus sequences were created with Geneious R7 (Biomatters Ltd., Auckland, New Zealand) or Sequencher 5.1 (Gene Codes, Ann Arbor, MI, USA). Sequences were double-checked by eye and aligned using ClustalW (Thompson et al. 1994) in BioEdit 7.2.5 (Hall 1999). Two phylogenetic analyses were performed using either only COI or all three regions (COI, 28S, ITS2). *Neuratelia minor* was used as an outgroup in all phylogenetic analyses.

For COI, a neighbour-joining tree implementing Kimura 2-parameter model (a standard model analysing DNA barcode data, see e.g. Waugh 2007, Õunap and Viidalepp 2009, Hausmann et al. 2013) was constructed in MEGA6 (Tamura et al. 2013). Clade credibilities were assessed by bootstrapping (1000 replications). The tree was visualised using MEGA6.

For the concatenated dataset, data were first divided into three subsets according to the markers used (COI, 28S and ITS). Thereafter, PartitionFinder 1.1.1 (Lanfear et al. 2012) was used to select the most effective partitioning scheme and best substitution model for each partition. Accroding to PartitionFinder results, COI and ITS were treated together as one partition keeping 28S separately for ML analysis with RAxML 7.7.1 (Stamatakis et al. 2008). A GTR+I substitution model was implemented on both partitions. Analysis was run using the default settings of the RAxML online platform (http://embnet.vital-it.ch/raxml-bb/index.php). Ten slow ML searches, one thorough ML search and 100 rapid boostrap replications were performed. The results of bootstrapping were drawn on a single best-scoring ML tree. Phylograms were visualised with FigTree v1.4.0 (http://tree.bio.ed.ac.uk/software/figtree/).

## Results

The morphology of studied material distinguished three previously known species of *Neuratelia* and a group of specimens, clearly delimited by characters of male terminalia. This group, represented by specimens from Estonia and Finland, resembles the widespread *Neuratelia nemoralis* (Meigen, 1818) and hereafter described and referred to as a new species – *Neuratelia salmelai* sp. n. In addition, another group of three specimens from different localities in Jabal Moussa Biosphere Reserve (Lebanon) had slight differences from *Neuratelia caucasica* Zaitzev, 1994 – a species only known from Caucasus. In the latter case, the species is described as *Neuratelia jabalmoussae* sp. n. but the morphological differences are diminutive underpinning the necessity of including DNA sequence data for species discrimination.

**Figure 4.**
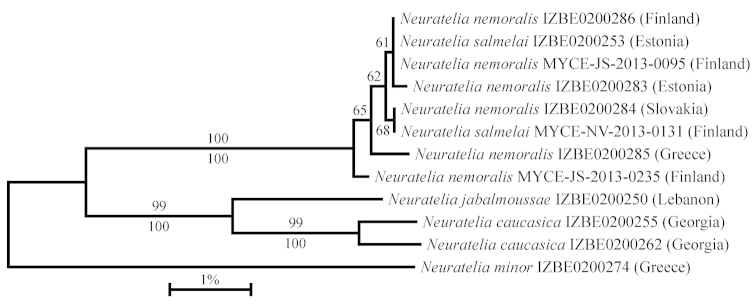
Neighbour-joining tree of the COI ’barcode’ region of *Neuratelia* spp. Scale bar: Kimura 2-parameter genetic distance. Bootstrap supports are presented above the branches. Maximum likelihood analysis of the concatenated (COI, 28S, ITS2) dataset yielded a tree with similar topology, bootstrap supports for the divergencies obtained in this analysis are given below the branches. Support values inferior to 60 are not shown.

Sequencing the ‘barcode region’ of COI was successful for all specimens included in the current study. The success rate was lower for ITS2 and 28S rDNA, as all attempts to sequence 28S failed for one individual of *Neuratelia nemoralis*, and for a few specimens, only half of 28S or ITS was obtained (Table 1). GenBank accession numbers for all sequences are presented in Table 1. The NJ tree constructed on the basis of barcodes divided the studied specimens into three well-supported clusters differing from each other by at least 4%. One group comprised only *Neuratelia caucasica* and another only *Neuratelia jabalmoussae*, whereas *Neuratelia nemoralis* and *Neuratelia salmelai* were intermingled in the third clade (Fig. 4). Studying the concatenated dataset resulted with almost identical results, as *Neuratelia jabalmoussae* and *Neuratelia caucasica* remained clearly separate sister taxa with *Neuratelia nemoralis* and *Neuratelia salmelai* remaining inseparable on the ML tree (Fig. 4).

**Table 1.** Voucher numbers, depositories and GenBank accession codes of studied *Neuratelia* specimens. *COI*: cytochrome oxidase subunit I; *28S*: 28S rRNA; *ITS2*: internal transcribed spacer 2.

Species	Voucher No	Depository	COI	28S	ITS2
*Neuratelia minor*	IZBE0200274	IZBE	KP715935	KP715924	KP715947
*Neuratelia nemoralis*	IZBE0200283	IZBE	KP715936	KP715925	KP715948
*Neuratelia nemoralis*	IZBE0200284	IZBE	KP715937	KP715926	KP715949
*Neuratelia nemoralis*	MYCE-JS-2013-0095	ZMUT	KP715938	KP715927	KP715950
*Neuratelia nemoralis*	IZBE0200286	IZBE	KP715939	×	KP715951
*Neuratelia nemoralis*	IZBE0200285	IZBE	KP715940	KP715928	KP715952
*Neuratelia nemoralis*	MYCE-JS-2013-0235	ZMUT	KP715941	KP715929	KP715953
*Neuratelia salmelai*	IZBE0200253	IZBE	KP715942	KP715930	KP715954
*Neuratelia salmelai*	MYCE-NV-2013-0131	ZMUT	KP715943	KP715931	KP715955
*Neuratelia jabalmoussae*	IZBE0200250	IZBE	KP715944	KP715932	KP715956
*Neuratelia caucasica*	IZBE0200255	IZBE	KP715945	KP715933	KP715957
*Neuratelia caucasica*	IZBE0200262	IZBE	KP715946	KP715934	KP715958

### The species

#### Neuratelia jabalmoussae sp. n.

Taxon classificationAnimaliaDipteraMycetophilidae

http://zoobank.org/95794D4E-8664-4BB5-80F2-762C125858BF

Figs 6 , 7 , 8 , 9 , 10 , 15

##### Type material.

*Holotype.* 1♂, LEBANON, Kesrouane Mar Elias, 34°03'06,9"N, 35°46'00,5"E, 1138 m a.s.l., at light, 27.v.–4.vi.2012, J. Kullberg leg. (IZBE0200250, slide mounted in Euparal with terminalia in glycerine). *Paratypes*. 1♂, LEBANON, Kesrouane Mar Geryes, 34°03'20,9"N, 35°44'28,9"E, 749 m a.s.l., at light, 26.v.–2.vi.2012, J. Kullberg leg. (IZBE0200251, in alcohol with terminalia in glycerine);1♂, LEBANON, Kesrouane Ghbele, 34°03'25,5"N, 35°43'02,5"E, 884 m a.s.l., at light, 26.v.–30.v.2012, J. Kullberg leg. (IZBE0200252, in alcohol).

##### Description.

**Male** (Fig. 6). Body length 5.4–5.8, 5.6 [5.6] mm (n=3).

**Figures 5–6.**
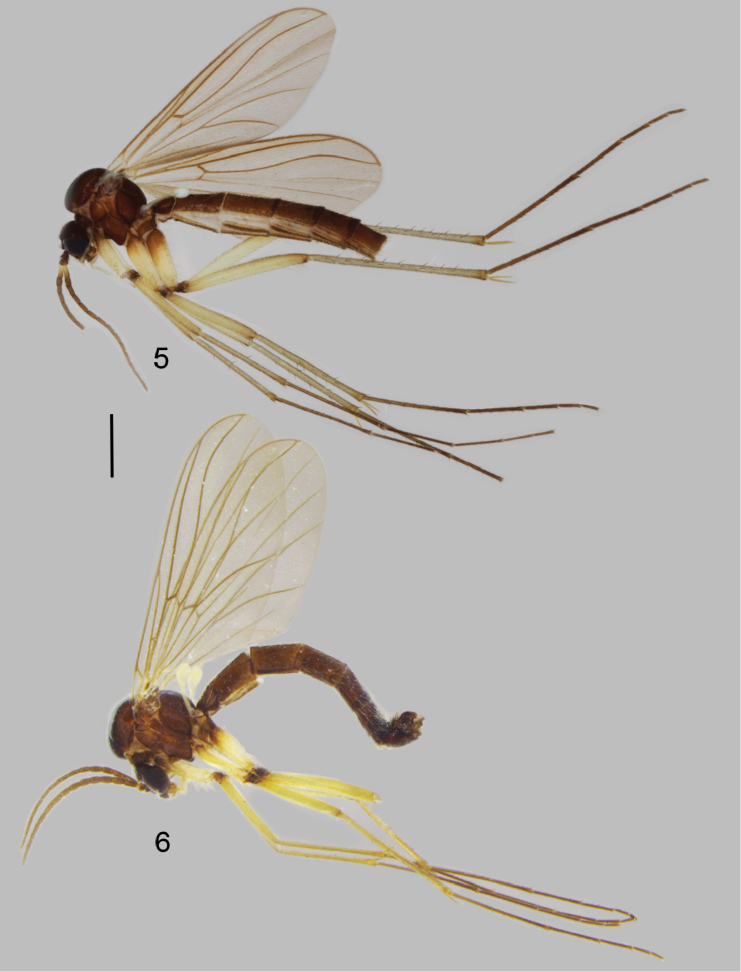
Habitus of *Neuratelia* species. **5** *Neuratelia salmelai* sp. n., terminalia detached **6** *Neuratelia jabalmoussae* sp. n. Scale bar = 1 mm.

**Head** dark brown, with numerous pale to yellowish setae. Three ocelli in a shallow and wide triangular arrangement, with laterals separated from eye margins by a distance slightly more than their own diameter. Face conical, about 0.8 times as wide as maximum height; clypeus rectangular, about 0.6 times as wide as high; both brown, setose, with setae on clypeus stronger than those on face. Mouthparts yellow. Palpus five segmented, yellowish with apex of fifth segment brownish. Ratios of three apical palpal segments 1.0: 1.68–1.85, 1.77 [1.85]: 1.97–2.00, 1.98 [2.00]. Scape and pedicel light brown to brown, flagellomeres light brown, with short yellowish setae. First flagellomere yellowish at basal third. Flagellum evenly tapering; first flagellomere clavate, 2.9–3.1 times as long as broad apically, 2–13 flagellomeres cylindrical, fourth flagellomere about 1.7–2.5 times as long as broad, apical flagellomere slightly conical, 4.8–5.4 times as long as broad at base.

**Thorax.** All parts brown to dark brown, all setae yellow to light brownish. Mesonotum with evenly arranged numerous setae. Scutellum wholly setose with about 10 stronger setae along the margin, not arranged to distinct pairs. Antepronotum with 8–9 and proepisternum with 4–7 setae of unequal size, laterotergite with 22–26 setae and mediotergite with ca 14–16 setae medially on lower part. Other pleural parts bare. Halteres pale yellow, setose.

**Legs.** All coxae yellow, basally infuscated. All trochanters brown. All femora and tibiae yellow, tarsi seem considerably darker because of dense setae. Foretibia with 1–2 ad, 0–2 d and 2–3 pd. Midtibia with 4–7 a, 2–3 d, 1–2 av and 2–4 pd. Hind tibia with 7–8 a, 1–2 ad (1 at apex), 4–6 d, 0–1 pd, 5 p and with a posterior apical comb of setae. Ratio of femur to tibia for fore-, mid- and hind legs: 0.86–0.94, 0.9 [0.9]; 0.77–0.86, 0.82 [0.77]; 0.71–0.75, 0.73 [0.75]. Ratio of tibia to basitarsus for fore-, mid- and hind legs: 0.91–0.94, 0.93 [0.94]; 1.26–1.3, 1.27 [1.3]; 1.51–1.54, 1,53 [1.51].

**Wing** hyaline, length 5.0–5.03, 5.02 [5.03] mm (n=3). All veins brown, costal and radial veins somewhat darker. Both surfaces of veins setose, except bare bM-Cu and r-m. Wing membrane with micro- and macrotrichia on both surfaces. Costa reaches very little from R_5_ to M_1_. Sc reaches costa at about one sixth between R_s_ and tip of R_1_. R_5_ sinuate. R_s_ about as long as crossvein r-m. M_1_ basally obsolete: observable vein begins distally from middle of R_1_. Cubital fork begins proximally from apex of Sc.

**Figures 7–8.**
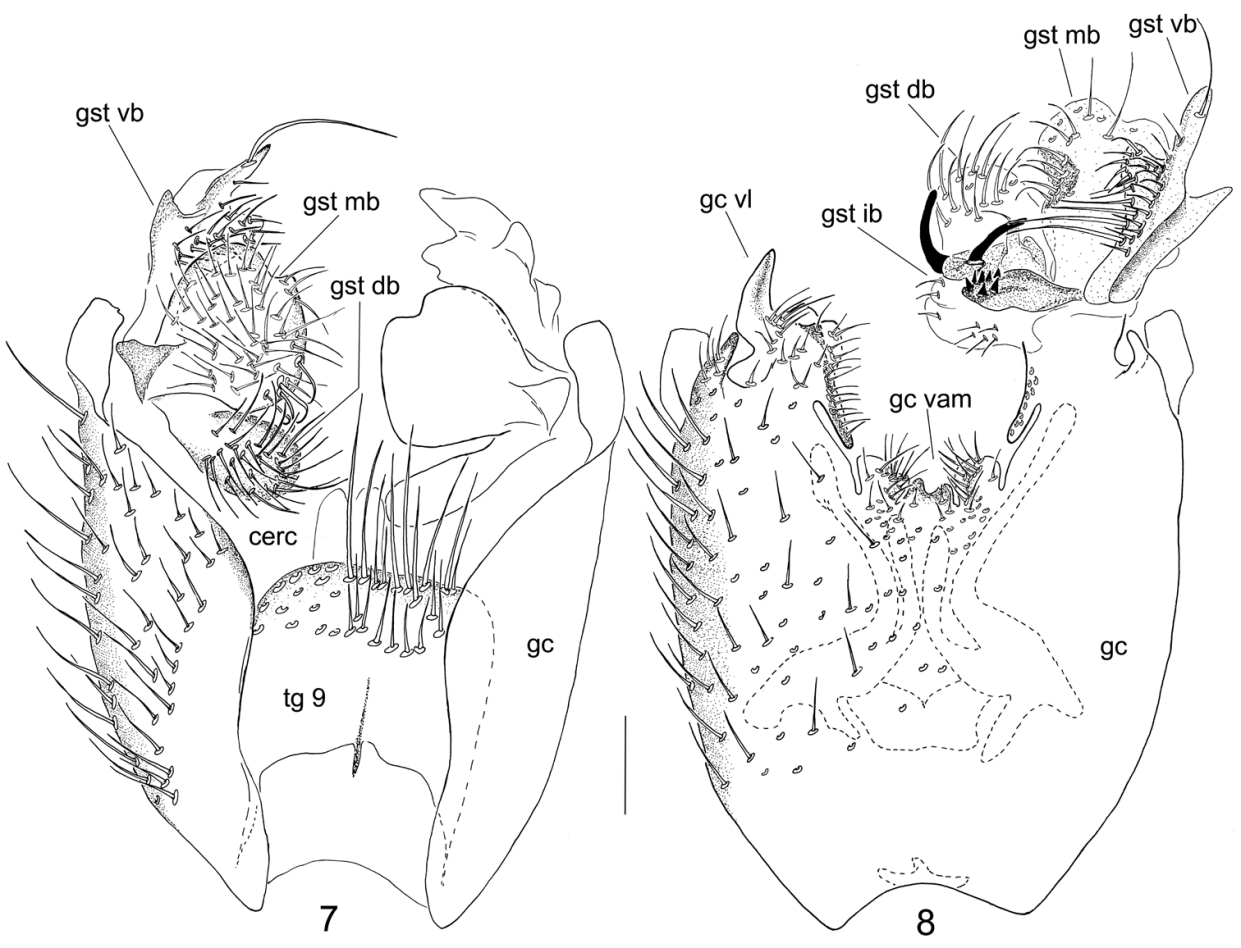
Male terminalia of *Neuratelia jabalmoussae* sp. n., dorsal view (**7**) and ventral view (**8**). Scale bar = 0.1 mm. Abbreviations: cerc = cerci; gc = gonocoxite; gc vam = ventroapical margin of gonocoxite; gc vl = ventroapical lobe of gonocoxite; gst db = dorsal branch of gonostylus; gst ib = internal branch of gonostylus; gst mb = medial branch of gonostylus; gst vb = ventral branch of gonostylus; tg 9 = IX tergite.

**Figures 9–14.**
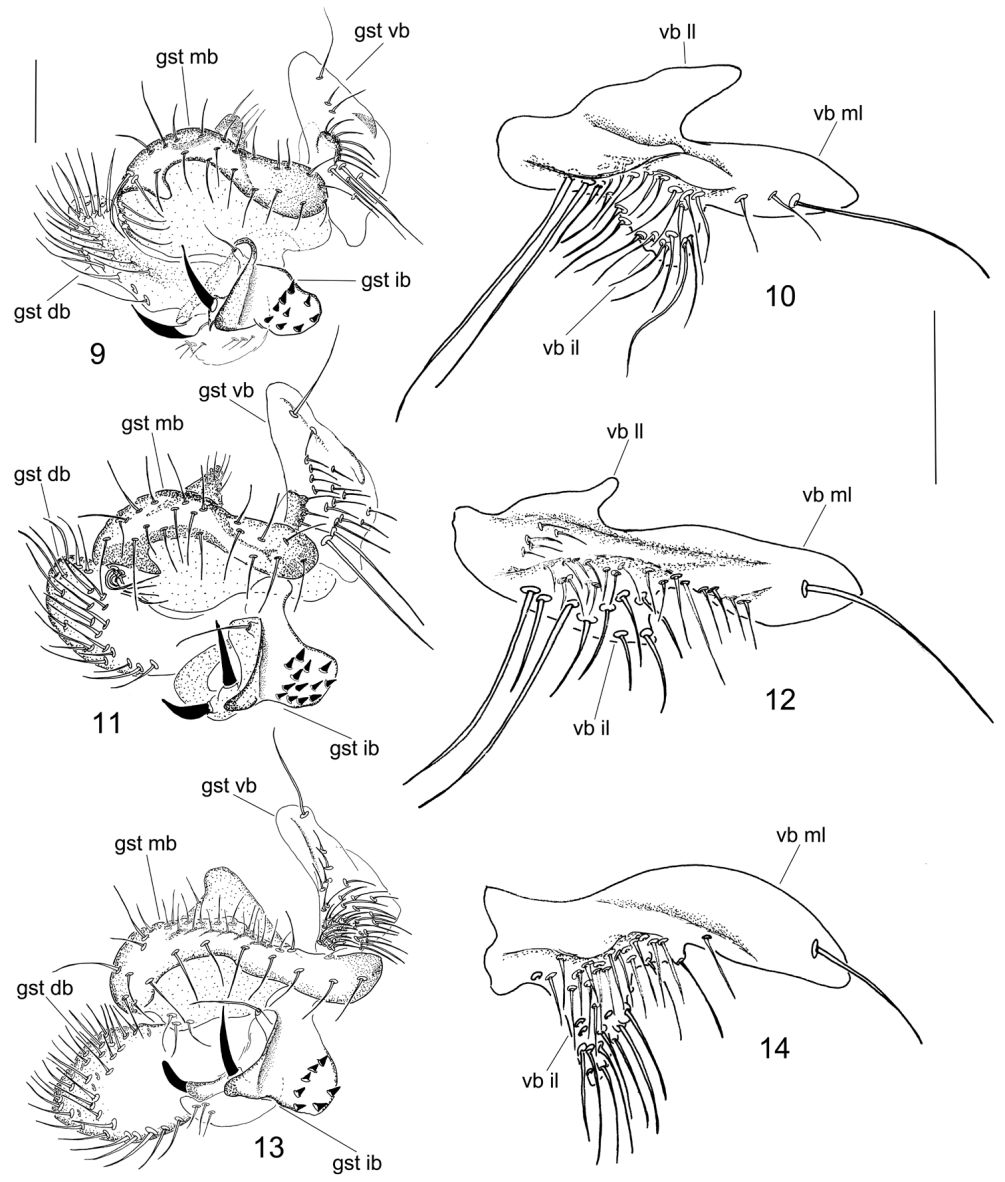
Male terminalia of *Neuratelia jabalmoussae* sp. n. (**9, 10**), *Neuratelia caucasica* Zaitzev, 1994 (**11, 12**) and *Neuratelia minor* (Lundström, 1912) (**13, 14**). **9**, **11**, **13** internal view of gonostylus **10**, **12**, **14** ventral view of ventral branch of gonostylus. Scale bars = 0.1 mm. Abbreviations: gst db = dorsal branch of gonostylus; gst ib = internal branch of gonostylus; gst mb = medial branch of gonostylus;gst vb = ventral branch of gonostylus; vb il = internal lobe of ventral branch of gonostylus; vb ll = lateral lobe of ventral branch of gonostylus; vb ml = medial lobe of ventral branch of gonostylus.

**Figures 15–17.**
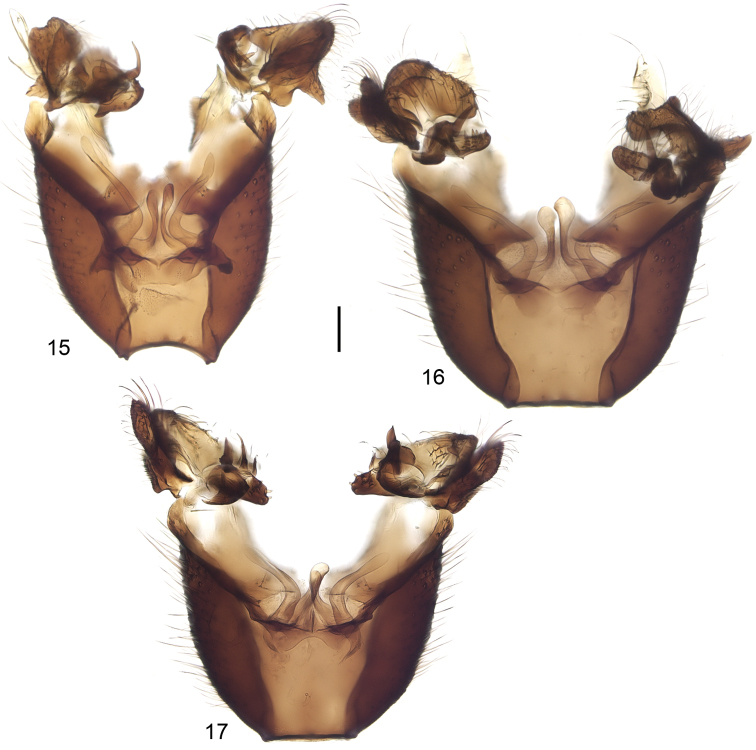
Male terminalia, dorsal view, tergite 9 removed. *Neuratelia jabalmoussae* sp. n. (**15**), *Neuratelia caucasica* Zaitzev, 1994 (**16**) and *Neuratelia minor* (Lundström, 1912) (**17**). Scale bar = 0.1 mm.

**Abdomen** with tergites brown and sternites yellowish. Tergites 6–7 somewhat darker. Terminalia (Figs 7, 8, 9, 10, 15) dark brown. Tergite 9 apically almost straight, with wide basal incision about one third of height of tergite. Basolateral portions of tergite 9 narrow and proximally pointed. Setae on tergite 9 similar to these on the gonocoxite, the posteriormost ones slightly stronger than the others. Cerci separated, protruding over tergite 9, with strong apical setae deviating from other setosity. The gonocoxite with a complex ventroapical lobe laterally; ventrobasally with wide shelving incision; ventroapical margin medially with lateral well delimited sub-circular and a medial apically concave setose structures. Dorsomedial margin of the gonocoxite slightly sinuous. The gonostylus with four branches. The dorsal branch simply oval, setose. The ventral branch trifid with 1) internal lobe setose including two stronger internally directed setae, 2) middle lobe elongated with a strong subapical seta, and 3) lateral lobe similar to internal lobe except being bare. Medial branch setose with a well delimited medial hump. Internal branch complex with two strong pointed spines and a lobe bearing 9 short spines ventrally on its apical part. The medial branch of the gonostylus connected with apical part of ventroapical lateral lobe of the gonocoxite. Parameres not protruding over ventroapical margin of gonocoxite.

**Female.** Unknown.

##### Biology.

Unknown.

##### Etymology.

The species is named after the type locality in Jabal Moussa Biosphere Reserve, Lebanon; the specific epithet is a noun in genitive case.

##### Specific discussion.

*Neuratelia jabalmoussae* sp. n. is very similar to *Neuratelia caucasica*, into which it also runs to in the key by Zaitzev (1994) because of having the foretibia slightly shorter than the fore basitarsus. Also the male terminalia of these two species are extremely similar, differ in details as follows: 1) paramers not expanded apically (Fig. 15), while they are well expanded in *Neuratelia caucasica* (Fig. 16), 2) the lateral lobe of the trifid ventral branch of the gonostylus prominent, about half of the size of medial lobe (Fig. 10), while it is minute in *Neuratelia caucasica*, about one fifth of the size of medial lobe (Fig. 12), and 3) internal branch of gonostylus has 8 short spines on a separate lobe (Fig. 9), while there are 13 spines in *Neuratelia caucasica* (Fig. 11). Both species share the general outline of male terminalia also with Western Palaearctic species *Neuratelia minor* (Lundström, 1912) and with *Neuratelia microdigitata* Sasakawa, 2004, known from Japan. However, *Neuratelia minor* has the foretibia slightly longer than fore basitarsus and the ventral branch of gonostylus bifid instead of being trifid. *Neuratelia microdigitata* has the internal branch of gonostylus with finger-like processes apically on a separate lobe (cf. Sasakawa 2004: fig. 4) instead of short spines as in other three species. All four species have the similar branching of the gonostylus and two strong pointed spines on internal branch of the gonostylus.

#### Neuratelia caucasica

Taxon classificationAnimaliaDipteraMycetophilidae

Zaitzev, 1994

Figs 11 , 12 , 16

##### Studied material.

GEORGIA. 2♂♂ 2♀♀, Surami, 42°01'34,2"N, 043°29'52,5"E, 941 m a.s.l., sweeping, O. Kurina leg. 18.v.2012 (IZBE0200255– IZBE0200258, in alcohol); 2♂♂ 1♀, Borjomi, 41°50'9,2"N, 043°19'56,7"E, 936 m a.s.l., sweeping, O. Kurina leg. 21.v.2012 (IZBE0200259, ♂, on pin with terminalia in glycerine; IZBE0200260, IZBE0200261, in alcohol); 3♂♂ 4♀♀, Mtirala NP, near visitor centre, 41°40'20,7"N, 041°52'31,8"E, 465 m a.s.l., sweeping, O. Kurina leg. 20.v.2013 (IZBE0200262, ♂, slidemounted in Euparal with terminalia in glycerine; IZBE0200263–IZBE0200268, in alcohol); 1♂ 2♀♀, Kintrishi NP, 41°45'11,7"N, 041°58'38,4"E, 453 m a.s.l., sweeping, O. Kurina leg. 22.v.2013 (IZBE0200269–IZBE0200271, in alcohol).

##### Specific discussion.

Having been described from North Caucasus (Krasnodarsk region), the species has not been recorded since and the studied material represents the first records from Georgia. According to male terminalia the species is close to *Neuratelia minor* and *Neuratelia jabalmoussae* sp. n.

#### Neuratelia minor

Taxon classificationAnimaliaDipteraMycetophilidae

(Lundström, 1912)

Figs 13 , 14 , 17

##### Studied material.

SLOVAKIA. 1♂, NP Muránska planina, Murán 3.5 km NE, sweeping, 48°45'46,5"N, 020°04'55,9"E, 483 m a.s.l. 30.v.2009, O. Kurina leg. (IZBE0200272, on pin with terminalia in glycerine; earlier published in Ševčík and Kurina 2011: 101); 1♂, NP Muránska planina, Šiance, sweeping, 48°46'14,7"N, 020°05'33,0"E, 656 m a.s.l. 30.v.2009, O. Kurina leg. (IZBE0200273, on pin with terminalia in glycerine; earlier published in Ševčík and Kurina 2011: 101). GREECE. 1♂, Central Macedonia, Kerkini lakes area, Vironia village, Beabies site, 41°19'15,4"N, 023°13'39,6"E, 1150 m a.s.l., Malaise trap, 19.–25.v.2008, G. Ramel leg. (IZBE0200274, slide mounted in Euparal).

##### Specific discussion.

*Neuratelia minor* was described and figured by Lundström (1912: figs 8, 9) from Romania. Because the type material was subsequently destroyed, Matile designated neotype from Hungary and provided also a new figure of male terminalia (Matile 1974: fig. 6). Both figures are sufficiently detailed, presenting a bifid ventral branch of the gonostylus that clearly discriminates the species morphologically from *Neuratelia caucasica* and *Neuratelia jabalmoussae* sp. n. *Neuratelia minor* has a more eastern distribution in the Western Palaearctic but is also found in France and the Eastern Palaearctic (Chandler 2013).

#### Neuratelia salmelai sp. n.

Taxon classificationAnimaliaDipteraMycetophilidae

http://zoobank.org/1554A8EF-A6FF-484D-9555-4855836A4263

**Figure 18.**
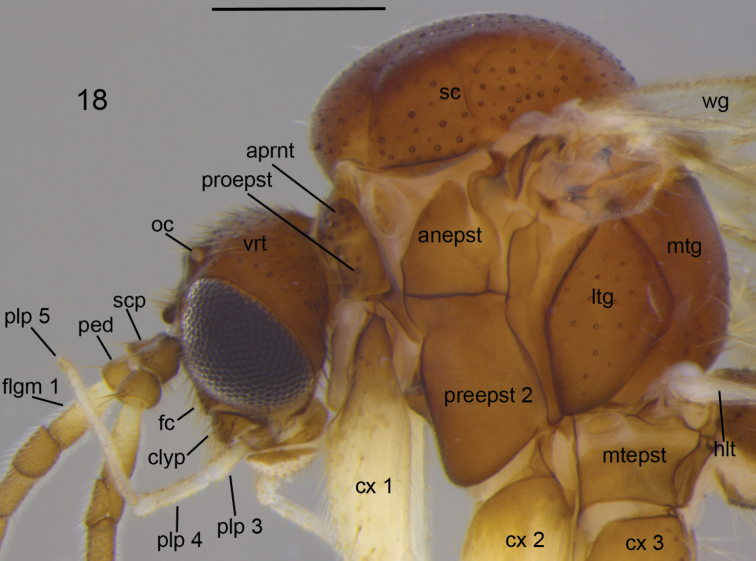
Head and thorax of *Neuratelia salmelai* sp. n. Scale bar = 0.5 mm. Abbreviations: anepst = anepisternum; aprnt = antepronotum; clyp = clypeus; cx = coxa; fc = face; flgm = flagellar segment; hlt = halter; ltg = laterotergite; mtepst = metepisternum; mtg = mediotergite; oc = ocellus; ped = pedicell; plp = palpal segment; preepst = preepisternum; proepst = proepisternum; sc = scutum; scp = scape; vrt = vertex; wg = wing.

Figs 5 , 18 , 20 , 22 , 24 , 26 , 28

##### Type material.

*Holotype.* 1♂, ESTONIA. Palupõhja, Kaha (ME 57), Malaise trap, 58°25'54,68"N, 026°14'28,90"E, 29.vi.–8.vii.2009, V. Soon leg. (IZBE0200253, slide mounted in Euparal with terminalia in glycerine). *Paratypes.* 1♂, FINLAND. Lkor: Sodankylä, Kaita-aapa, Malaise trap, 67°50'45,5"N, 026°33'17,6"E, 5.vi.–3.vii.2012, J. Salmela leg. (IZBE0200254, in alcohol with terminalia in glycerine); 1♂, FINLAND. Lkoc: Kittilä, Kielisenpalo, Malaise trap, 68°01'16,6"N, 025°03'46,9"E, 26.vi–24.vii.2007, J. Salmela leg. (MYCE-NV-2013-0093 in ZMUT, in alcohol with terminalia in glycerine); 1♂, FINLAND. Lkoc: Kittilä, Vuotsonperän-jänkä, Malaise trap, 67°37'15,9"N, 025°26'43,6"E, 25.vi.–24.vii.2009, J. Salmela leg. (MYCE-NV-2013-0131 in ZMUT, in alcohol with terminalia in glycerine); 1♂, FINLAND, Lkor: Sodankylä, Pomokaira 67°52'19,2"N, 026°12'46,8"E, 11.6.-10.7.2013, J. Salmela leg. Malaise trap Salix swamp with seepages (DIPT-JS-2014-0199 in JSPC, in alcohol).

##### Description.

**Male** (Figs 5, 18). Body length 5.8–6.5, 6.2 [5.8] mm (n=4).

**Head** (Fig. 18) brown to dark brown, with numerous setae. Three ocelli in a shallow and wide triangular arrangement, with laterals separated from eye margins by a distance about twice of their own diameter. Face conical, about 0.9 times as wide as maximum height; clypeus rectangular, about 0.6 times as wide as high; both brown, setose, with setae on clypeus stronger than those on face. Mouthparts light brown. Palpus five segmented, yellowish brown with second segment and apex of fifth segment darker. Ratios of three apical palpal segments 1.0: 1.37–1.65, 1.52 [1.56]: 1.62–1.82, 1.72 [1.71]. Scape and pedicel brown, pedicel somewhat lighter; flagellomeres light brown, with short pale setae. First flagellomere yellowish at basal half. Flagellum evenly tapering; first flagellomere clavate, 2.5–3.3 times as long as broad apically, 2–13 flagellomeres cylindrical, fourth flagellomere about 2.3–2.6 times as long as broad, apical flagellomere slightly conical, 5.2–6 times as long as broad at base.

**Thorax** (Fig. 18). All parts brown to dark brown, all setae yellow to light brownish. Mesonotum with evenly arranged numerous setae. Scutellum with about 10 setae along the margin, not arranged to distinct pairs. Antepronotum with 10–13 and proepisternum with 5–8 setae of unequal size, laterotergite with 17–26 setae and mediotergite with ca 12–20 setae medially on lower part. Other pleural parts bare. Halteres yellow, setose.

**Legs.** All coxae yellow with basal fourths brown. In case of two paratypes, cx_3_ entirely light brownish with darker basal half. All trochanters brown. All femora and tibiae yellow, tarsi seem darker because of dense setae. Foretibia with 2–3 ad, 1–3 d and 2–3 pd. Midtibia with 6–10 a, 0–4 d, 4–5 av and 2–3 pd. Hind tibia with 8–10 a, 1–2 ad (1 at apex), 7–8 d, 5–7 p and with a posterior apical comb of setae. Ratio of femur to tibia for fore-, mid- and hind legs: 0.86–0.91, 0.88 [0.91]; 0.72–0.87, 0.80 [0.87]; 0.72–0.77, 0.75 [0.72]. Ratio of tibia to basitarsus for fore-, mid- and hind legs: 0.81–1.00, 0.9 [0.9]; 1.22–1.33, 1.27 [1.22]; [1.66].

**Wing** hyaline, length 4.1–5.0, 4.52 [4.49] mm (n=4). All veins brown, costal and radial veins somewhat darker. Both surfaces of all veins setose. Wing membrane with micro- and macrotrichia on both surfaces. Costa reaches very little from R_5_ to M_1_. Sc reaches costa at about quarter between R_s_ and tip of R_1_. R_5_ sinuate. R_s_ about as long as crossvein r-m. M_1_ basally obsolete: observable vein begins distally from middle of R_1_. Cubital fork begins proximally from apex of Sc.

**Abdomen** with tergites brown to dark brown and with sternites yellow to brownish yellow. Terminalia (Figs 20, 22, 24, 26, 28) dark brown. Tergite 9 apically rounded, with deep and narrow basal incision about half of height of tergite. Basolateral portions of tergite 9 tapering. Setae on tergite 9 similar to these on the gonocoxite. Cerci fused, protruding over tergite 9, with strong apical setae deviating from other setosity. The gonocoxite with elongated dorsoapical and ventroapical lobes. Dorsoapical lobe of the gonocoxite dorsobasally right-angled and apically tapering, both well exposed in lateral view and with subapical medially directed hump. Dorsomedial margin of the gonocoxite slightly undulating. Ventroapical lobe of the gonocoxa apically rounded and subapically somewhat deformed. The gonocoxite ventrobasally with wide shelving incision and ventroapically well sclerotised, with a medial cleft. The gonostylus with two branches. The dorsal branch kidney-shaped, slightly widening towards medial line. The ventral branch elongated, apically evenly rounded, with a clear medial widening which bears strong setae well deviating from other setosity of the branch. The medial widening of the ventral branch of gonostylus connected with apical part of the ventroapical lobe of gonocoxite. Parameres long, sinuous, protruding over ventroapical margin of gonocoxite.

**Figures 19–22.**
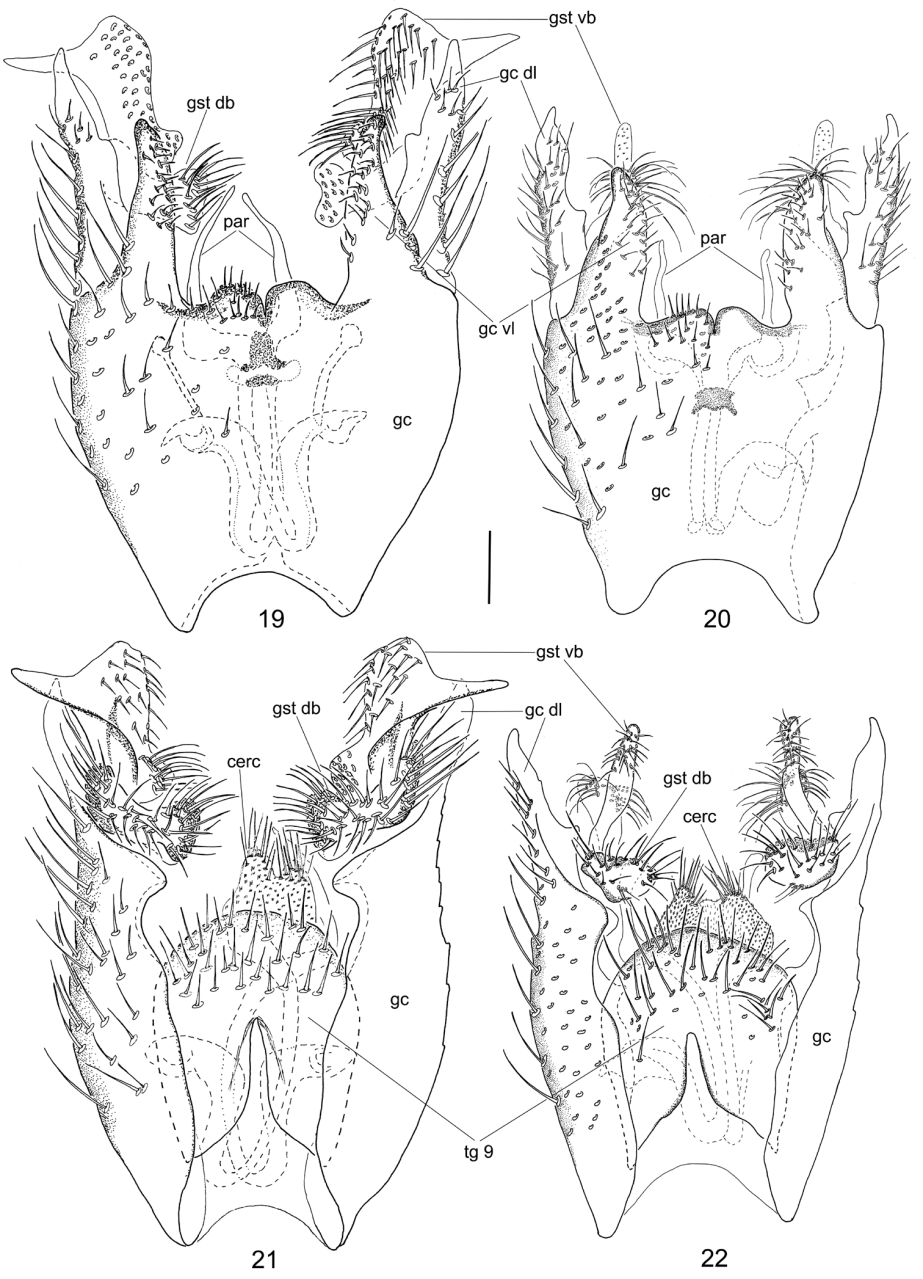
Male terminalia of *Neuratelia nemoralis* (Meigen, 1818) (**19**, **21**) and *Neuratelia salmelai* sp. n. (**20**, **22**). Ventral view (**19**, **20**) and dorsal view (**21**, **22**). Scale bar = 0.1 mm. For abbreviations: see Figs 7–8, except: gc dl = dorsoapical lobe of gonocoxite; par = parameres.

**Figures 23–26.**
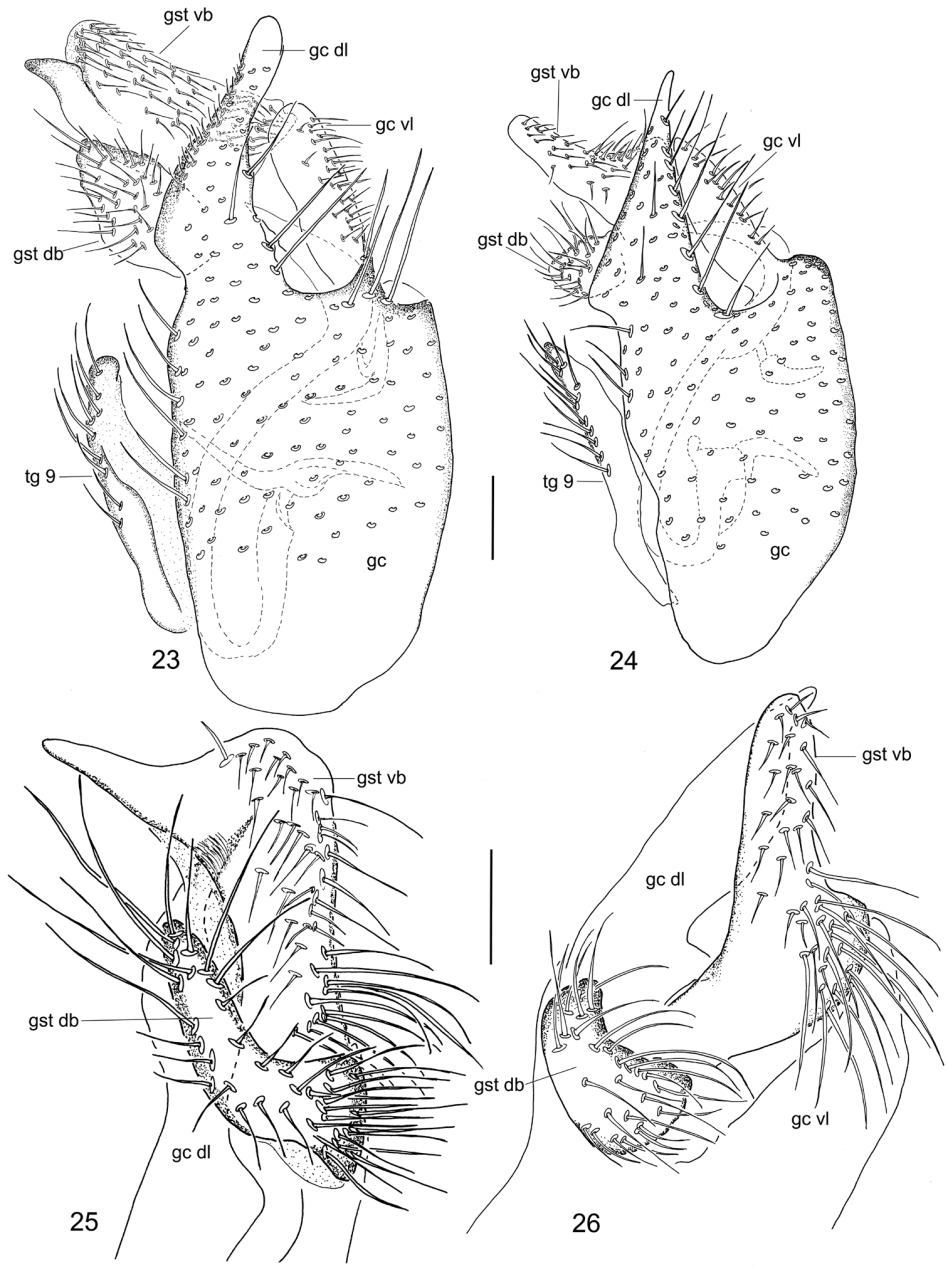
Male terminalia of *Neuratelia nemoralis* (Meigen, 1818) (**23**, **25**) and *Neuratelia salmelai* sp. n. (**24**, **26**). Lateral view (**23**, **24**) and internal view of the gonostylus (**25**, **26**). Scale bar = 0.1 mm. For abbreviations: see Figs 7–8 and 19–22.

**Figures 27–28.**
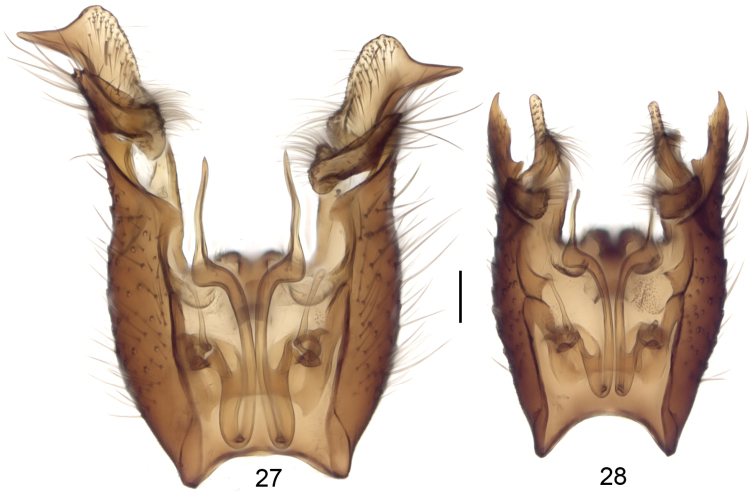
Male terminalia, dorsal view, tergite 9 removed. *Neuratelia nemoralis* (**27**) and *Neuratelia salmelai* sp. n. (**28**). Scale bar = 0.1 mm.

**Female.** Unknown.

##### Biology.

Unknown.

##### Etymology.

The species is named in honour of Dr. Jukka Salmela, who kindly provided us the material collected from Finland.

##### Specific discussion.

Following the key by Zaitzev (1994) the new species runs to *Neuratelia sintenisi* Lackschewitz, 1937, as its foretibia is usually shorter than fore-basitarsus. However, this character seems to be variable, as these are of equal length in one paratype, guiding to *Neuratelia nemoralis*, a species with so far reported Holarctic distribution (Laffoon 1965, Zaitzev 1994, but see specific discussion under the latter). In sharing the general outline of male terminalia, *Neuratelia salmelai* resembles in addition to *Neuratelia nemoralis* also to *Neuratelia kamijoi* Sasakawa, 2004 from Japan. All three species have gonocoxite with protruding lobes dorsoapically and ventroapically, and two-branched gonostylus. *Neuratelia kamijoi* has the dorsoapical lobe of gonocoxite with clear subapical tooth (cf. Sasakawa 2004: fig. 5) while in the other two species it is more simple. *Neuratelia salmelai* differs markedly from *Neuratelia nemoralis* as follows: 1) dorsoapical lobe of gonocoxite apically tapering (apically evenly rounded in *Neuratelia nemoralis*), 2) dorsal branch of gonostylus kidney-shaped and slightly widening towards medial line (elongated, curved and sharply widening towards medial line in *Neuratelia nemoralis*), and 3) ventral branch of gonostylus apically evenly rounded with medial widening that bears strong setae deviating well from other setosity of the lobe (ventral branch of gonostylus apically pointed with subapical widening that bears normal setae not deviating from other setosity of the branch in *Neuratelia nemoralis*).

#### Neuratelia nemoralis

Taxon classificationAnimaliaDipteraMycetophilidae

(Meigen, 1818)

Figs 19 , 22 , 23 , 25 , 27

##### Studied material.

FINLAND. 1♂, Lkor: Savukoski, Törmäoja, Malaise trap, 67°50'48,5"N, 029°28'20,8"E, 14.vi–10.vii.2012, J. Salmela leg. (MYCE-JS-2013-0095 in ZMUT, in alcohol with terminalia in glycerine); 1♂, Lkor: Sodankylä, Tarmpomapää, Malaise trap, 67°59'14,0"N, 025°55'09,4"E, 1.–29.ii.2009, J. Salmela leg. (MYCE-JS-2013-0235 in ZMUT, in alcohol with terminalia in glycerine); 1♂, Ab: Turku, Pomponrahka, Malaise trap, 2011, J. Salmela leg., (IZBE0200286, in alcohol with terminalia in glycerine). ESTONIA. 2♂♂, Matsalu NP, Matsalu village, window trap, 58°44'04"N, 023°42'42"E, 29.v–17.vi.2009, I. Süda leg. (IZBE0200275, IZBE0200276, in alcohol); 7♂♂, Palupõhja, Kaha (ME 57), Malaise trap, 58°25'54,68"N, 026°14'28,90"E, 31.v–15.vi.2009, V. Soon leg. (IZBE0200277– IZBE0200282, in alcohol; IZBE0200283, slide mounted in Euparal). SLOVAKIA. 1♂, NP Slovenský raj, Javorina Mt., sweeping, 48°53'23,1"N, 020°15'20,8"E, 1112 m a.s.l., 4.vi.2009, O. Kurina leg (IZBE0200284, in alcohol with terminalia in glycerine; earlier published in Ševčík and Kurina 2011: 101). GREECE. 1♂, Central Macedonia, Kerkini lakes area, Vironia village, Beabies site, 41°19'15,4"N, 023°13'39,6"E, 1150 m a.s.l., Malaise trap, 19.–25.v.2008, G. Ramel leg. (IZBE0200285, in alcohol with terminalia in glycerine).

##### Specific discussion.

This is a widely distributed species in the Palaearctic region (Zaitzev 1994, Sasakawa 2004, Chandler 2013), and as far as we know supposed to extend also to North America (Laffoon 1965, Chandler 2013). The species was first reported from the Nearctic region by Coquillett (1900: 391) and thereafter by Johannsen (1911: 264, Fig. 145) and Fisher (1937: 171, Plate 12: Fig. 12), while all subsequently published information is of secondary nature. However, Fisher (1937: 171) already questioned conspecificity of the Nearctic material and as far as we can judge from the figures of both authors, these represent a different species. Thus, the occurrence of *Neuratelia nemoralis* in the Nearctic region remains open with need for the future study.

## Discussion

This study combines for the first time the results of morphological and molecular analyses for delineating species of fungus gnats. As a common practice in insect taxonomy, we relied on characters of male genitalia and the mitochondrial COI barcoding, respectively. In one case, however, these two types of data provided conflicting evidence for species delimitation in the fungus gnat genus *Neuratelia*. Therefore, additional characters were sought by sequencing also the 28S and ITS2 regions of the nuclear ribosomal DNA. The latter is becoming increasingly applicable in delimitation of insect taxa (e.g. Rokas et al. 2002, Wilkerson et al. 2004, Haarto and Ståhls 2014). While COI has successfully been used in studies on fungus gnat taxonomy (Martinsson et al. 2011, Rindal et al. 2009b, Ševčík et al. 2013, 2014) and ecology (Põldmaa et al. 2015), ITS2 has been incorporated only in a few studies (Ševčík et al. 2013, 2014).

Taxonomic work on insects has mostly been carried out on the basis of morphological examination. In many cases where studying external characters fail to yield unequivocal results, genital morphology has been proven to be valuable source of additional information (Hosken and Stockley 2004). In more complicated cases, however, even the most detailed morphological study can remain inconclusive. One of the ‘classic’ scenarios where morphological examination may produce questionable results is allopatry. Thus, solving the taxonomic status of morphologically similar allopatric populations has for long time been one of the key questions for systematists. There has been no clear practice how to handle such cases, as acquisition of diagnostic characters does not always happen in the same order or at the same rate for different groups of organisms (e. g. Mutanen et al. 2012). Morphological study of *Neuratelia caucasica* and *Neuratelia jabalmoussae* presents one more case following the ‘classic’ scenario: these species have clearly separate geographic ranges located no less than a thousand kilometres away from each other but their morphological differences are minute. However, the genetic distance (quantified according to the Kimura 2-parameter model) calculated from the COI barcoding region is only 1.5% between the two specimens of *Neuratelia caucasica*, but ranges from 4.0% to 4.3% between that species and *Neuratelia jabalmoussae* (Fig. 4). Many studies have shown that intraspecific genetic distance in the barcode region is several times smaller than the interspecific genetic distance (e. g. Hausmann et al. 2011, Huemer et al. 2014, Pentinsaari et al. 2014). Average intraspecific genetic distance remains under 1% in different insect orders, with only few known exceptions (e. g. Hebert et al. 2010, Park et al. 2011, Pentinsaari et al. 2014). The 4% difference between *Neuratelia caucasica* and *Neuratelia jabalmoussae* exceeds usual intraspecific genetic variation in insects for more than 4 times, suggesting these taxa truly are different species. This conclusion is further corroborated by few substitutions and small length variation, both in the ITS2 and 28S regions of rDNA distinguishing the two species. The three gene regions thus provide evidence for considering *Neuratelia caucasica* and *Neuratelia jabalmoussae* to represent distinct species.

The situation with *Neuratelia salmelai* and *Neuratelia nemoralis* is, however, much more intriguing. Regarding these species, there are five COI barcode haplotypes in our data matrix that have a maximum 1% genetic distance. Specimens of *Neuratelia salmelai* and *Neuratelia nemoralis* are impossible to distinguish from each other on the basis of barcode data, as the holotype of *Neuratelia salmelai* from Estonia carries COI sequence that is identical to that of two specimens of *Neuratelia nemoralis* from two different regions of Finland. A Finnish paratype of *Neuratelia salmelai*, on the other hand, has COI sequence identical to that of a specimen of *Neuratelia nemoralis* from Slovakia. Such a situation has been called ‘barcode sharing’ in literature (e.g. Hausmann et al. 2011, 2013). Additionally, the relatively rapidly evolving sequences of nuclear rDNA 28S and ITS2, did not allow us to distinguish *Neuratelia salmelai* and *Neuratelia nemoralis*. 28S was identical in all studied specimens of both species, whereas one individual of *Neuratelia nemoralis* from Greece had ITS2 haplotype identical to that of both specimens of *Neuratelia salmelai*, which differ from the remaining specimens of *Neuratelia nemoralis* by one substitution in ITS2. Thus, delineating *Neuratelia salmelai* from *Neuratelia nemoralis* on the basis of current molecular data is not possible regardless of whether a distance-based or character-based (DeSalle et al. 2005) approach is selected.

In contrast to the failure of genetic markers to distinguish *Neuratelia salmelai* and *Neuratelia nemoralis*, their male terminalia were remarkably different. The differences are more pronounced than among the other three species included in this study. Most likely these taxa represent recently diverged species that still share the genetic diversity of their common ancestor. The evolution of insect genitalia can be more rapid than diversification of commonly studied markers (Raupach et al. 2010, Hausmann et al. 2013). Another possibility, hybridisation between females of *Neuratelia nemoralis* and males of some other fungus gnat species, deserves less credit for at least two reasons. First, though interspecific hybridisation sometimes occurs in closely related insects, hybrids usually are confined to clear hybrid zones or exist in particular sympatric populations (Mallet et al. 2011, Sánchez-Guillén et al. 2014). Therefore they constitute only a small proportion of the total population. In the current case the five males of *Neuratelia salmelai* are 26% of the 19-individual sample of *Neuratelia nemoralis* group in our study. This is an unrealistically high proportion for hybrids, as material was randomly collected from different parts of these species’ ranges, not concentrating on a particular region where hybridisation could occur. Second, if there really had been hybridisation, it would be natural to assume that putative hybrids (i. e. *Neuratelia salmelai*) share genetic material with specimens collected from geographically close localities. This is not the case, as no geographic pattern was detected when collecting localities of *Neuratelia salmelai* and *Neuratelia nemoralis* were taken into account. Apparently, the *Neuratelia salmelai*/*Neuratelia nemoralis* species pair is one of the rare occasions where nucleotide data from common markers and morphological characters do not corroborate each other. Large-scale barcoding projects have shown that such cases usually constitute no more than one or two per cents of the total diversity of insects (e. g. Mutanen et al. 2012, Huemer et al. 2014, Pentinsaari et al. 2014).

## Supplementary Material

XML Treatment for Neuratelia jabalmoussae

XML Treatment for Neuratelia caucasica

XML Treatment for Neuratelia minor

XML Treatment for Neuratelia salmelai

XML Treatment for Neuratelia nemoralis
